# Oxygen for relief of dyspnoea in mildly- or non-hypoxaemic patients with cancer: a systematic review and meta-analysis

**DOI:** 10.1038/sj.bjc.6604161

**Published:** 2008-01-08

**Authors:** H E Uronis, D C Currow, D C McCrory, G P Samsa, A P Abernethy

**Affiliations:** 1 Division of Medical Oncology, Department of Medicine, Duke University Medical Center, Durham, NC, USA; 2 Health Services Research and Development, Durham Veteran's Affairs Medical Center, Durham, NC, USA; 3 Department of Palliative and Supportive Services, Division of Medicine, Flinders University, Bedford Park, South Australia, Australia; 4 Division of General Internal Medicine, Department of Medicine, Duke University Medical Center, Durham, NC, USA; 5 Center for Clinical Health Policy Research, Duke University Medical Center, Durham, NC, USA; 6 Department of Biostatistics and Bioinformatics, Duke University, Durham, NC, USA

**Keywords:** neoplasms (MeSH), dyspnoea (MeSH), palliative care (MeSH), oxygen (MeSH)

## Abstract

The aim of this study was to determine the efficacy of palliative oxygen for relief of dyspnoea in cancer patients. MEDLINE and EMBASE were searched for randomised controlled trials, comparing oxygen and medical air in cancer patients not qualifying for home oxygen therapy. Abstracts were reviewed and studies were selected using Cochrane methodology. The included studies provided oxygen at rest or during a 6-min walk. The primary outcome was dyspnoea. Standardised mean differences (SMDs) were used to combine scores. Five studies were identified; one was excluded from meta-analysis due to data presentation. Individual patient data were obtained from the authors of the three of the four remaining studies (one each from England, Australia, and the United States). A total of 134 patients were included in the meta-analysis. Oxygen failed to improve dyspnoea in mildly- or non-hypoxaemic cancer patients (SMD=−0.09, 95% confidence interval −0.22 to 0.04; *P*=0.16). Results were stable to a sensitivity analysis, excluding studies requiring the use of imputed quantities. In this small meta-analysis, oxygen did not provide symptomatic benefit for cancer patients with refractory dyspnoea, who would not normally qualify for home oxygen therapy. Further study of the use of oxygen in this population is warranted given its widespread use.

Dyspnoea is common, affecting 50–70% of patients with advanced cancer (Reuben and Mor, 1986; Ripamonti, 1999; Bruera *et al*, 2000). Defined by the American Thoracic Society as ‘a subjective experience of breathing discomfort that consists of qualitatively distinct sensations that vary in intensity’, dyspnoea is a very personal experience (ATS, 1999). Descriptions vary widely; examples include ‘short of breath’, ‘hard to move air’, ‘chest tightness’, ‘choking’, ‘panting’, and ‘gasping’ (Brown *et al*, 1986; Simon *et al*, 1989; Roberts *et al*, 1993; Harver *et al*, 2000). The experience of dyspnoea can be affected by many conditions, including the cancer itself, coexisting diseases, and cancer cachexia (Reuben and Mor, 1986; Ripamonti, 1999; Bruera *et al*, 2000). Dyspnoea in cancer survivors has also been correlated with psychological status, including both anxiety and depression (Bruera *et al*, 2000; Tanaka *et al*, 2002a), and over 20% of cancer patients report interference with psychological functioning (Tanaka *et al*, 2002b).

The sensation cannot always be explained by organic causes (Ahmedzai, 1998; Bruera and Ripamonti, 1998) and is influenced by pathways and interactions at multiple levels of the nervous system (Bruera *et al*, 2000). Dyspnoea appears to be a subjective sensation that is not a direct representation of the intensity of the stimulus in the nervous system but rather the result of an interaction among production, perception, and expression (Ripamonti and Bruera, 1997; Bruera and Ripamonti, 1998).

Management of dyspnoea presents a challenge because there is no roadmap to guide therapy. The typical recommendation is to relieve dyspnoea by treating the underlying cause, but this is often not successful or simply not possible in people with advanced cancer. In these cases, dyspnoea is termed ‘refractory’ (Abernethy *et al*, 2003) and the focus is on symptom control in an effort to decrease the sensation of dyspnoea. Clinicians choose from a number of palliative interventions, including opioids, psychotropic agents, and nebulised furosemide.

There are data on the role of oxygen in changing survival in hypoxaemic patients (P_a_O_2_<55 mmHg) with COPD (chronic obstructive pulmonary disease) (NOTTG, 1980; MRCWP, 1981). Data regarding the role of oxygen in relieving the sensation of breathlessness are inconclusive. Evidence for the use of oxygen to relieve the sensation of dyspnoea, the so-called ‘palliative oxygen’, in patients with malignancy, is also lacking. Despite this, use of palliative oxygen to relieve breathlessness toward the end of life is supported by consensus guidelines (Kvale *et al*, 2003; Storey and Knight, 2003; Booth *et al*, 2004) and is a common practice. For example, a recent survey of 648 palliative-care specialists and respiratory physicians in Australia and New Zealand demonstrated that palliative oxygen is commonly prescribed with 58% of 214 respondents reporting a belief that palliative oxygen is beneficial and 65% reporting that the most common reason for prescribing oxygen was refractory dyspnoea (Abernethy *et al*, 2005). Canadian physicians report similar practices (Stringer *et al*, 2004).

The discrepancy between clinical practice and available evidence has several implications. First, patients may be prescribed ineffective treatments. Additionally, oxygen is not a benign intervention. Quality of life may be limited as a result of functional restriction from tubing, tanks, or concentrators; there may be psychological distress in being reliant on a machine (Currow *et al*, 2007); nasal cannulae might irritate the nose and increase the risk of epistaxis. Home oxygen therapy is also expensive. If patients do not meet funding criteria for home oxygen, they must pay out-of-pocket or receive the intervention on compassionate-use grounds. Funding for home oxygen therapy is a common reason for referral to hospice care. In Canada, about 40% of patients receiving home oxygen do not meet funding guidelines and receive this intervention on a compassionate-use basis (Guyatt *et al*, 2000).

In an attempt to improve the understanding of the optimal use of palliative oxygen in patients with malignancy, we conducted a systematic review and meta-analysis aimed at answering the following question: ‘in mildly hypoxaemic or non-hypoxaemic cancer patients with breathlessness, does oxygen therapy improve symptoms?’

## METHODS

### Definitions and outcomes

Oxygen administered by a non-invasive method was defined as oxygen delivered by nasal cannula, mouthpiece, or face mask. Studies that evaluated the effects of oxygen on dyspnoea, either at rest or on exertion, as measured by patient self-report, were sought. Secondary outcomes of interest included quality of life, patient preference, and functional status.

### Search strategy

A general search aimed at identifying articles evaluating the use of oxygen in the context of breathlessness was identified by exploding the MeSH terms *dyspnoea*, *oxygen*, and *oxygen inhalation therapy* and combining them. The text words *oxygen*, *dyspnoea*, and *breathlessness* were also included. The article set generated by this search was then combined with a standard search for randomised controlled trials (Dickersin *et al*, 1994). The strategies were executed in MEDLINE and EMBASE (from 1966 to December 2006), and were limited to articles involving adult human beings and published in English. Reference lists of included studies and relevant systematic reviews were hand searched.

### Literature screening

Abstracts and full-text versions of articles identified in MEDLINE, EMBASE, and other searches were screened by two investigators (HU and AA) against the following eight exclusion criteria:

- Study design was not a randomised controlled trial;
- Study subjects were not adults with malignancy;
- Study subjects had a mean P_a_O_2_<55 mmHg or more than 50% of subjects had oxygen saturation <88% by pulse oximetry;
- Study subjects were already receiving home oxygen therapy;
- Study intervention was not oxygen *vs* placebo;
- Method of oxygen delivery was something other than nasal cannula, mouthpiece, or mask;
- No dyspnoea outcomes were reported; and,
- ‘Other’ reason (e.g., study articles were not editorial or review article).

All abstracts were reviewed by two oncologists (HU and AA) and any article selected by either reviewer was included for full-text review. All full-text articles were reviewed by both oncologists (HU and AA), and the full-text articles meeting all inclusion criteria were selected for full abstraction. Differences in judgment were resolved by consensus conference.

### Data abstraction

For each article meeting the inclusion criteria, basic study parameters were abstracted into evidence tables summarising the following: study design, primary focus, inclusion/exclusion criteria, interventions and how administered, subjects, outcomes, results, and quality assessment. Abstractions were performed by one investigator (HU) and were over-read by a second investigator (AA) to ensure accuracy.

### Quality assessment

Studies were assessed for both internal and external validity. Internal validity criteria included randomisation, blinding, and description of withdrawals/dropouts, and each study was assigned a Jadad score (Jadad *et al*, 1996). External validity criteria included subject description, detailed intervention description, and adequately reported dyspnoea outcomes.

### Data analysis

Dyspnoea ratings measured by modified Borg, 0–10 numerical rating scale (NRS), 100 mm visual analog scale (VAS), or 300 mm VAS were converted into standardised mean differences (SMDs). Results of both periods of crossover trials were used. Crossover trials should be included in meta-analyses using results from paired analyses. However, these data are often not available. In these cases, standard errors were estimated using methods described by Follmann *et al* (1992). Correlations between repeated outcomes were estimated from *P*-values when available and, when unavailable, the lowest estimate from other studies was used.

Statistical analyses, except meta-analyses, were conducted using SAS E-Guide version 3 for Windows (SAS Institute, Cary, NC, USA). Meta-analyses were conducted for those studies where means and variances for dyspnoea measurements could be estimated from published reports. Authors were contacted for additional data if published information was not sufficient. For meta-analyses, effect sizes were calculated using Cochrane software, RevMan 4.2.8., and are reported as SMD with 95% confidence intervals (CIs). Two-sided *P*-values are reported and statistical significance was assumed if *P*<0.05 (Supplementary Information).

## RESULTS

### Article review

**Figure 1:**
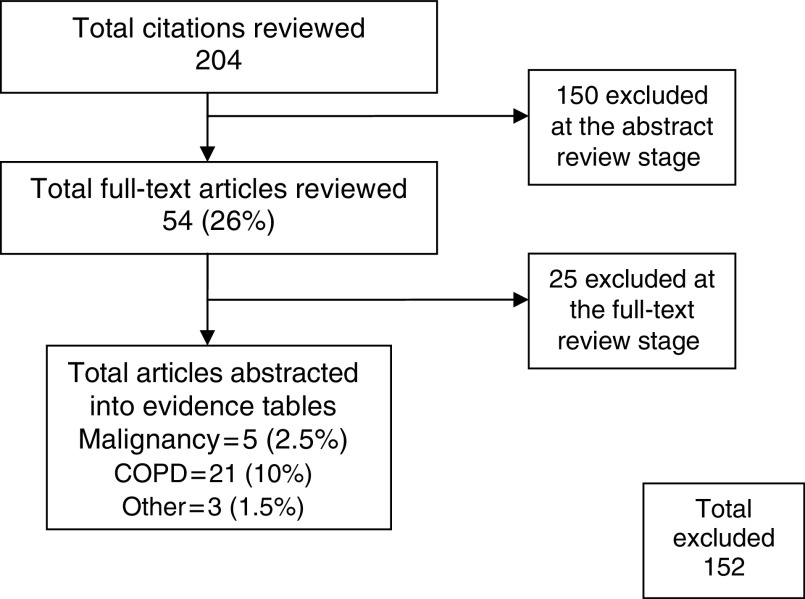
Flowchart of articles reviewed for the systematic analysis of the benefit of palliative oxygen for the relief of dyspnoea in people with cancer who do not qualify for long-term domiciliary oxygen therapy.

The flow of articles reviewed is presented in Figure 1. This study on cancer was conducted in conjunction with a second review that focused on COPD and other aetiologies of breathlessness. Studies that did not include cancer patients were excluded from this analysis. The five abstracted articles represent data from five different studies (Table 1; Bruera *et al*, 1993, 2003; Booth *et al*, 1996; Ahmedzai *et al*, 2004; Philip *et al*, 2006).

### Study characteristics

#### Design characteristics

All five studies included in this analysis were blinded, randomised, and controlled crossover trials. In this type of trial, participants are assigned to study arms consisting of two or more treatments given consecutively and in random order (Sibbald and Roberts, 1998). Each subject acts as his/her own control, so that the response to treatment A can be compared with that of treatment B without concern for issues involving patient variation, if the duration of therapy in each arm means that the other factors affecting the symptom are likely to remain stable for the entire study. Crossover trials are commonly used in palliative-care research given the ability to directly compare two treatments in the same patient and the ability to answer a clinical question with fewer subjects than is required in a traditional parallel group trial. As is typical for palliative-care studies, sample sizes in the five studies included were small with a median of 33 participants per study and a mean of 29.6 (s.d. 16.5). None of the studies included had evidence of calculations required to claim adequate power to answer the questions.

#### Patient characteristics

The five included studies represented 148 participants, all of them adults. Median participant age was 65 and 39% were females; no information was available on race or ethnicity. Participants had several different types of malignancy, with lung cancer (65%) or unspecified cancer with metastasis to the lung (15%) being most common. The profile of other malignancies was as follows: breast (5%), colon (3%), and others, including lymphoma, melanoma, sarcoma, carcinoid, skin, bladder, and head and neck (7%). Baseline percent oxygen saturation that was provided by four out of five studies are as follows: median 93% (Philip *et al*, 2006), median 94% (Ahmedzai *et al*, 2004), median 98% (Bruera *et al*, 2003), and range 80–99% (Booth *et al*, 1996). Baseline dyspnoea at rest that was provided in three out of five studies are as follows: 0 mm by modified Borg (Ahmedzai *et al*, 2004), 5 mm by NRS (Bruera *et al*, 2003), and 59 mm by 100 mm VAS (Booth *et al*, 1996).

#### Intervention characteristics

Four studies (Bruera *et al*, 1993, 2003; Booth *et al*, 1996; Philip *et al*, 2006) were focused on evaluating oxygen *vs* medical air for relief of dyspnoea, whereas the fourth evaluated the use of Heliox28, a novel agent containing 72% helium and 28% oxygen *vs* oxygen and medical air (Ahmedzai *et al*, 2004). Oxygen was delivered by nasal canula in three studies (Booth *et al*, 1996; Bruera *et al*, 2003; Philip *et al*, 2006) and by mask in two (Bruera *et al*, 1993; Ahmedzai *et al*, 2004); doses of oxygen ranged from 3 to 5 l min^−1^. Oxygen was administered at rest in three studies (Bruera *et al*, 1993; Booth *et al*, 1996; Philip *et al*, 2006) and during a 6MWT (6-min walk test) in two studies (Bruera *et al*, 2003; Ahmedzai *et al*, 2004).

### Treatment efficacy

#### Estimation of treatment efficacy

**Figure 2:**
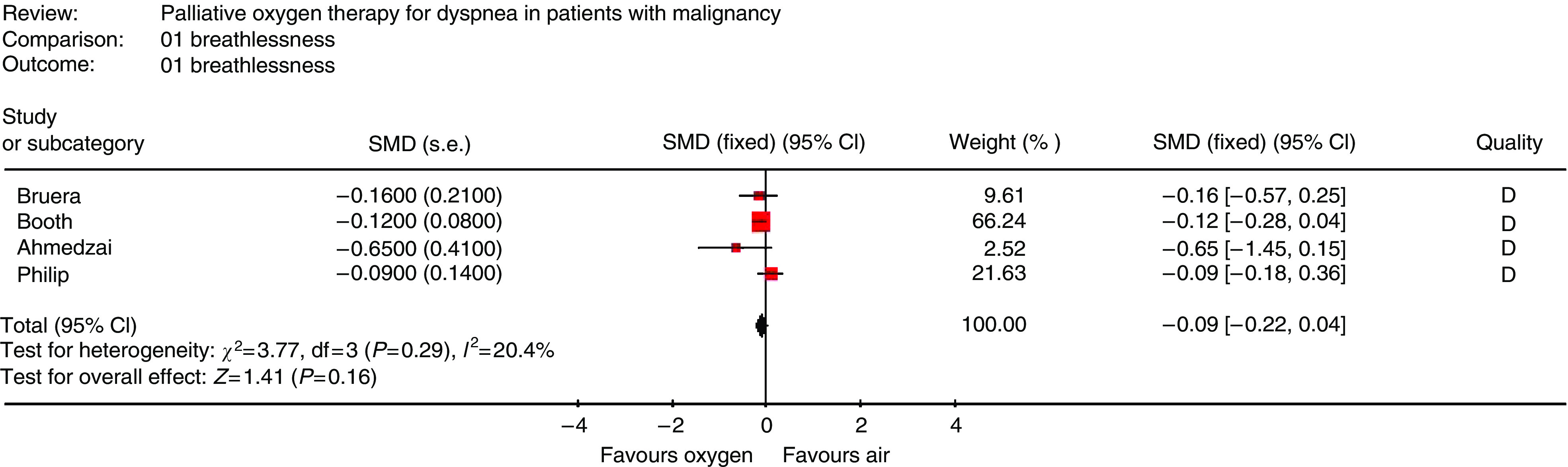
Estimation of efficacy of oxygen in the treatment of dyspnoea in cancer patients who do not qualify for long-term domiciliary oxygen therapy.

**Figure 3:**
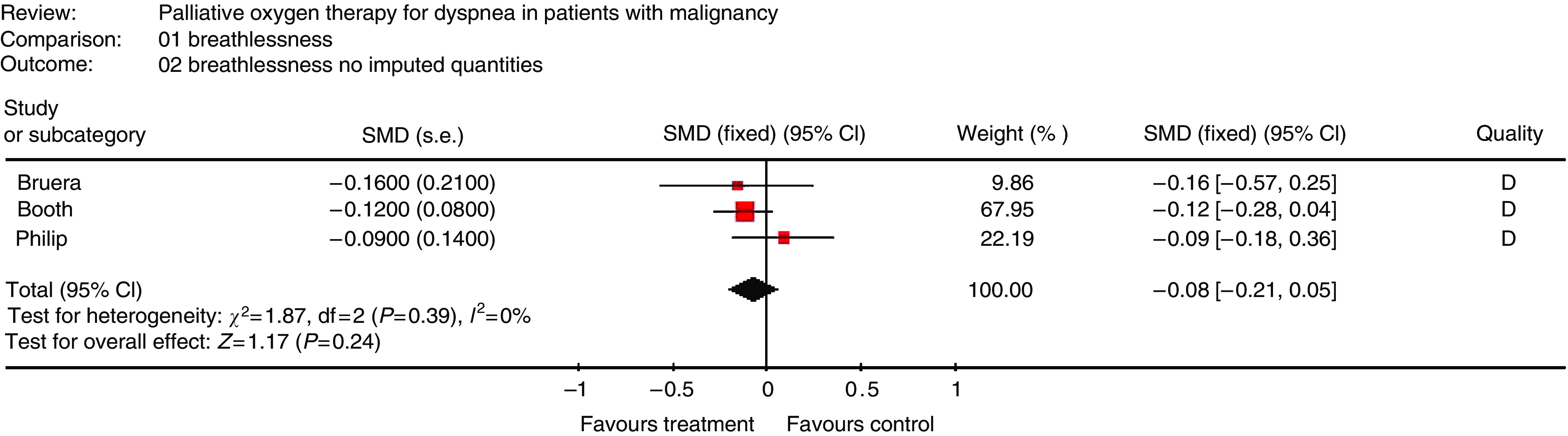
Sensitivity analysis of blinded, randomised controlled trials exploring the symptomatic benefit of oxygen therapy in reducing refractory dyspnoea in a palliative population which does not qualify for domiciliary oxygen – no studies requiring use of imputed quantities.

Overall, the quality of reporting was poor with four out of five studies having a Jadad score of 2, indicating inadequate discussion of methods of both randomisation and blinding (Jadad *et al*, 1996). Only one out of five studies provided sufficient information to calculate SMD and variances for dyspnoea measurements. As a result, all the authors were contacted to request individual patient data. All the authors responded; individual patient data were available for three studies (Booth *et al*, 1996; Bruera *et al*, 2003; Philip *et al*, 2006) and were unavailable for the other two (Bruera *et al*, 1993; Ahmedzai *et al*, 2004). Using correlations calculated from available data, it was possible to derive s.e. for a fourth study (Ahmedzai *et al*, 2004). The remaining study (Bruera *et al*, 1993) was excluded from meta-analysis, leaving 134 patients included. Oxygen failed to improve dyspnoea in mildly- or non-hypoxaemic cancer patients (SMD=−0.09, 95% CI −0.22 to 0.04; *P*=0.16) (Figure 2). This finding is consistent with a global assessment reflecting that four out of five individual studies were negative. Sensitivity analysis excluding the study requiring the use of imputed quantities was performed; results were stable in this analysis (Figure 3).

#### Other outcomes

Two out of five included studies also reported the impact of oxygen on results of the distance walked during the 6MWT (Bruera *et al*, 2003; Ahmedzai *et al*, 2004). One study reported a statistically significant increase in the distance with the use of oxygen (174.6 m (s.d. 11.2)) *vs* medical air (128.8 m (s.d. 10.3)) (*P*<0.01) (Ahmedzai *et al*, 2004). However, a second study reported no difference in distance with oxygen (331.6 m (s.d. 54.9)) and with medical air (330.7 (s.d. 57.6)) (Bruera *et al*, 2003). Four out of five studies reported results of still-blinded patient preference for oxygen *vs* medical air. Two out of four studies reported a statistically significant still-blinded patient preference for oxygen *vs* air, whereas the other two found no such difference (Table 2).

## DISCUSSION

Oxygen was not effective at reducing the sensation of dyspnoea in cancer patients who would not otherwise qualify for home oxygen therapy, with an SMD=−0.09 (95% CI: −0.22 to 0.04; *P*=0.16) translating into a 0.19-cm reduction in dyspnoea on a 10 cm VAS or a 0.22-point reduction in dyspnoea on a 0–10 NRS. When evaluating interventions for dyspnoea, most clinicians would consider a change of 10 cm on a 10 cm VAS or a 1-point reduction on a 0–10 NRS to be clinically significant. So, the observed reduction does not represent a clinically significant change.

Two out of five studies evaluated the impact of oxygen on exercise tolerance by evaluating patients during a 6MWT conducted either with oxygen or medical air. The results were conflicting with one study finding a clinically and statistically significant improvement in distance walked with oxygen as opposed to air and the other failing to do so. Why might this be the case? It is not immediately clear because the two populations were very similar. In the study by Bruera *et al* (2003), median age was 64 years, 64% were male, 94% had primary lung cancer, median baseline oxygen saturation was 98%, and median usual dyspnoea on activity (as measured by 0–10 NRS) was 5. In the study by Ahmedzai *et al* (2004), median age was 72.5 years, 58% were male, 100% had primary lung cancer, median baseline oxygen saturation was 94%, and median dyspnoea on exertion (as measured by modified Borg) was 3. The similarities between the two samples suggest that the conflicting results are likely due to chance and that further study is warranted.

Patient preference was also discussed in two out of four studies. This is known to be important as the COPD literature has demonstrated that even patients shown to get benefited from oxygen do not always wish to receive it (Eaton *et al*, 2002; Currow *et al*, 2007). It is also a critical issue given the subjective nature of dyspnoea and the fact that patients often have difficulty in describing the sensation (ATS, 1999). Unfortunately, the data on patient preference are not definitive, with only two out of four studies demonstrating statistically significant patient preference for oxygen. Again, the reason for this lack of agreement is not clear, but it may be a result of small sample size and/or relative heterogeneity in the study population. Interestingly, one of the studies reporting a significant patient preference for oxygen (Bruera *et al*, 2003) failed to find a statistically significant improvement in dyspnoea as measured by the 0–10 NRS. This may reflect the subjective nature of dyspnoea as well as the difficulties in measuring the sensation, particularly in cancer patients.

Our systematic review does have several strengths. First, the search was conducted in both MEDLINE and EMBASE, allowing incorporation of a broad range of publications and increasing our ability to identify articles addressing this specific question. Second, we contacted the authors of the included studies for additional data. All responded and individual patient data were subsequently available for an additional three studies, significantly strengthening our meta-analysis. Third, we followed Cochrane methodology for study review, data abstraction, and data analysis.

In addition to the inherent issues associated with the current body of literature evaluating the optimal use of oxygen in dyspnoeic cancer patients who did not qualify for the home oxygen therapy, there are several limitations specific to our review. First, patients were included in each of the studies based on oxygen saturation as measured by pulse oximetry, a reasonably accurate way of evaluating oxygenation but one that is affected by a number of physiologic variables, including haemoglobin level, 2,3-diphosphoglycerate levels, arterial blood flow, temperature of the digit where the oximeter is located, skin pigmentation, and motion artifact (Jensen *et al*, 1998). Although direct measurement of arterial oxygen saturation by arterial blood gas analysis would be preferable, the use of pulse oximetry is not surprising given that these studies were performed in a palliative-care population where invasive testing is typically minimised. Second, the patient population represents a mixture of individuals with oxygen saturations both above and below 90% by pulse oximetry. While we set out to exclude studies evaluating patients who were clearly hypoxaemic (P_a_O_2_<55 mmHg), it became clear that this would not be possible due to the lack of arterial blood gas data. We attempted to decrease the impact on our analysis by excluding those studies where more than 50% of participants had a baseline oxygen saturation of <90% (roughly equivalent to a P_a_O_2_ of 60 mmHg using the oxyhaemoglobin dissociation curve) from meta-analysis; no studies meeting this criterion were identified. As a result, we may have included individuals with P_a_O_2_<55 mmHg and/or excluded individuals with P_a_O_2_=55–59 mmHg. Both scenarios could certainly impact our analysis although there are no available data to suggest the direction in which that impact would be seen. Third, the sample size was small (*n*=148) and 65% of patients carried a diagnosis of lung cancer. This limits the generalizability of these results to a population of patients with a wider range of malignancies given the number of factors that contribute to dyspnoea in this population.

In summary, available data do not provide support for the use of palliative oxygen for relief of the sensation of refractory dyspnoea in cancer patients. However, limitations in the data make it difficult to come to firm conclusions on such an important issue as evidenced by the fact that clinicians across the globe continue to prescribe oxygen for refractory dyspnoea (Abernethy *et al*, 2005). The data on patient preference, obtained when patients were still-blinded to the intervention, suggest that there is a population of patients who experience less dyspnoea while receiving oxygen as compared with medical air. Further research is required to appropriately identify these subgroups of people. Until such data are available, decisions regarding the use of palliative oxygen can be made on an individual basis after an ‘*n* of 1’ assessment as described by Bruera *et al* (1992).

## Figures and Tables

**Table 1:** Characteristics of included studies exploring the role of oxygen therapy in people with refractory dyspnoea who do not qualify for long-term oxygen therapy

**Reference**	***n***	**Population**	**O_2_ saturation <90% included?**	**Intervention**	**Outcome measure**	**Results**	**Quality^a^**
(Philip *et al*, 2006)	51	Cancer of any type, dyspnoea	Yes 17 (33%)	CA *vs* O_2_, 4 l min^−1^ at rest	100 mm VAS	No significant difference in dyspnoea with O_2_ *vs* CA	2
(Ahmedzai *et al*, 1998, 2004)	12	Lung cancer, dyspnoea on exertion	No	CA *vs* O_2_, 8–10 l min^−1^ during 6MWT	Modified Borg and 100 mm VAS	No significant difference in dyspnoea with O_2_ *vs* CA	2
(Bruera *et al*, 2003)	33	Advanced cancer of any type, dyspnoea at rest or on mild exertion	No	CA *vs* O_2_, 5 l min^−1^ during 6MWT	NRS	No significant difference in dyspnoea with O_2_ *vs* CA	5
(Booth *et al*, 1996, 2004)	38	Advanced cancer of any type, dyspnoea at rest	Yes 6 (16%)	CA *vs* O_2_, 4 l min^−1^ at rest	Modified Borg and 100 mm VAS	No significant difference in dyspnoea with O_2_ *vs* CA	2
(Bruera *et al*, 1993)	14	Advanced cancer of any type, dyspnoea, oxygen saturation < 90%	Yes 14 (100%)	CA *vs* O_2_ 5 l min^−1^ at rest	NRS	Significant improvement in dyspnoea with O_2_ *vs* CA	2

Abbreviations: CA=compressed air; O_2_=oxygen; 6MWT=6-min walk test; VAS=visual analog scale; NRS=numerical rating scale.

a Quality as assessed by Jadad score (Jadad *et al*, 1996).

**Table 2:** Blinded patient preference in the four randomised controlled studies that were assessing the role of oxygen in relieving refractory dyspnoea in people who currently do not qualify for long-term domiciliary oxygen therapy

**Reference**	***n***	**Population**	**Patient preference**
(Philip *et al*, 2006)	51	Cancer of any type, dyspnoea	Preference for oxygen=21 (41%)
			Preference for air=15 (29%)
			No preference=15 (29%)
			
(Ahmedzai *et al*, 1998, 2004)	12	Lung cancer, dyspnoea on exertion	Not reported
			
(Bruera *et al*, 2003)	33	Advanced cancer of any type, dyspnoea at rest or on mild exertion	Preference for oxygen=19 (58%)^a^
			Preference for air=11 (33%)^a^
			No preference=3 (9%)
			
(Booth *et al*, 1996, 2004)	38	Advanced cancer of any type, dyspnoea at rest	Preference for oxygen=15 (54%)^b^
			Preference for air=11 (39%)^b^
			Worse with oxygen=2 (7%)^b^
			Worse with air=3 (11%)^b^
			
(Bruera *et al*, 1993)	14	Advanced cancer of any type, dyspnoea, oxygen saturation <90%	Preference for oxygen=12 (86%)^c^
			Preference for air=1 (7%)^c^
			No preference=1 (7%)^c^

^a^*P*<0.05 for comparison.

^b^Based only on those who made some comment (*n*=28).

^c^*P*<0.001 for comparison.
