# Supplementary material for: Oxygen for relief of dyspnoea in mildly- or non-hypoxaemic patients with cancer: a systematic review and meta-analysis
Source: Br J Cancer. 2008 Jan 8;98(2):294–9. doi: 10.1038/sj.bjc.6604161 (PMC2361446; doi:10.1038/sj.bjc.6604161)
Supplement: Supplementary Information [file 6604161x1.doc]

**APPENDIX I**

Traditional parallel trials randomize individuals to one of two treatments and serve as the model for the randomized, double-blind, controlled trials often held up as the strongest level of evidence. Cross-over trials, on the other hand, are trials in which each individual receives two or more treatments in a *sequence that is random*[1]. Each subject acts as his/her control so any response to treatment can be compared without concern for patient variation. Use of this design allows one to answer research questions with fewer individuals than would be required for a traditional parallel trial. Because smaller sample sizes are adequate, cross-over trials are very commonly used in palliative care research where is it often difficult to enroll patients. Additionally, because patients receive both treatments under evaluation, they are able to report any personal preference for one treatment over another. Appropriate analysis of cross-over trials involves the use of a method specific to paired data (such as the paired *t*-test for parametric data or McNemar’s test for non-parametric data)[2].

Performing a systematic review and meta-analysis that includes cross-over trials poses different challenges, mostly due to data presentation. It is easiest to use data that is reported as within-patient differences (i.e. the mean difference between the outcome after Treatment A and after Treatment B) because this allows one to calculate the standard deviation (SD) and 95% confidence interval (CI) directly. However, data from cross-over trials is seldom presented this way. Instead, data is commonly presented as two separate means (i.e. the mean (SD) for treatment A and the mean (SD) for treatment B). In this case, the mean difference between treatments is derived by subtraction as follows[2]:

mean Treatment A – mean Treatment B = mean difference for Formula 1

Treatment A vs. Treatment B (MD)

This set of means may or may not be accompanied by a p-value. If a reported p-value is the result of a paired analysis, its correlating t-statistic can be used to determine the standard error of the difference (SE_diff_) using the following formula[2]:

SE_diff_ = MD / t Formula 2

The SD of the mean difference (SD_diff_) can then be calculated as follows[2]:

SD_diff_ = SE_diff_ [sqrt (n)] Formula 3

Finally, the correlation between the two measurements can be calculated from the following formula[2]:

SD (A) ^2^ + SD (B) ^2^ – SD_diff_^2^

ρ = _________________________ Formula 4

2 x SD (A) x SD (B)

SD (A) = standard deviation of the mean for treatment A and SD (B) is standard deviation of the mean for treatment B

When including studies that measure outcomes with more than one measure, one needs to calculate a standardized mean difference (SMD) as the measure of effect. The formula is as follows[2]:

SMD = MD / pooled SD Formula 5

pooled SD = sqrt [SD (A) ^2^ + SD (B) ^2^ / 2]

The variance of the SMD is calculated as follows[3]:

Variance = 2 x (1 – ρ) / n Formula 6

ρ is from Formula 4

n = number of subjects in trial

Data is also presented in such a way that one cannot use it at all. In these cases, as long as means are available, one can use the lowest ρ (see Formula 4) from the other included studies to calculate the variance using Formula 6. If data is presented only as medians, the study must be excluded from meta-analysis.

The procedure outlined above (using Formulas 1 – 6) was used to calculate the SMD used in the Forest plot for studies included in this meta-analysis. As mentioned, individual patient data was available for both Booth et al. and Bruera et al. The variance for the SMD for Ahmedzai et al. was calculated using Formula 6 with ρ = 0.58, a value derived from another systematic review and meta-analysis of the use of oxygen versus air for breathlessness in COPD patients. Effect size was estimated using the generic inverse ratio method utilized by Cochrane software, RevMan 4.2.8, using a fixed effects approach.

**REFERENCES**

1 Sibbald B, Roberts C. Understanding controlled trials. Crossover trials. ***BMJ*** 1998;316:1719.

2 Elbourne DR, Altman DG, Higgins JP, Curtin F, Worthington HV, Vail A. Meta-analyses involving cross-over trials: methodological issues. ***Int J Epidemiol*** 2002;31:140-149.

3 Follmann D, Elliott P, Suh I, Cutler J. Variance imputation for overviews of clinical trials with continuous response. ***J Clin Epidemiol*** 1992;45:769-773.
